# BlinkLinMulT: Transformer-Based Eye Blink Detection

**DOI:** 10.3390/jimaging9100196

**Published:** 2023-09-26

**Authors:** Ádám Fodor, Kristian Fenech, András Lőrincz

**Affiliations:** Department of Artificial Intelligence, Eötvös Loránd University, Pázmány Péter stny 1/A, 1117 Budapest, Hungary; foauaai@inf.elte.hu (Á.F.); lorincz@inf.elte.hu (A.L.)

**Keywords:** eye blink detection, classification, deep learning, multimodal fusion, transformers

## Abstract

This work presents BlinkLinMulT, a transformer-based framework for eye blink detection. While most existing approaches rely on frame-wise eye state classification, recent advancements in transformer-based sequence models have not been explored in the blink detection literature. Our approach effectively combines low- and high-level feature sequences with linear complexity cross-modal attention mechanisms and addresses challenges such as lighting changes and a wide range of head poses. Our work is the first to leverage the transformer architecture for blink presence detection and eye state recognition while successfully implementing an efficient fusion of input features. In our experiments, we utilized several publicly available benchmark datasets (CEW, ZJU, MRL Eye, RT-BENE, EyeBlink8, Researcher’s Night, and TalkingFace) to extensively show the state-of-the-art performance and generalization capability of our trained model. We hope the proposed method can serve as a new baseline for further research.

## 1. Introduction

Detecting eye blinks is a challenging problem that can be used to solve a number of facial analysis tasks; it can reveal deep fake manipulation, detect driver drowsiness, measure the level of attention and eye fatigue during task performance, and health disorders, among others. Most approaches depend on face recognition and frame-wise eye state classification, even with the rise of transformer-based sequence models in many other research areas. However, these methods may struggle to handle cases where blinks occur for only a few frames or where input data are noisy or incomplete.

The machine learning and computer vision communities are focused on using different visual representations to tackle the blinking detection problem. Three categories emerged in the last decade: (i) feature-based methods use predetermined higher-level features like iris and eye landmark distances, (ii) appearance-based methods utilize low-level eye representations (e.g., RGB texture) and then adopt learning algorithms to classify eye states, and (iii) motion-based methods use input representations that encode motion information or a sequence of eye-related features to determine eye states.

### 1.1. Feature-Based Methods

Chen et al. [1] proposed an eye blink detection and gaze estimation method. After the eyes are detected, several image preprocessing procedures are performed to eliminate the noise caused by the changes in normal-light conditions and reflections: pixel gradients, valley-peak field, fitted parabolas, iris mask, and pupil center landmark are calculated, among others. Using an adaptive Starburst extraction algorithm, their proposed technique correctly identifies both the iris and limbus features in various lighting conditions, and then the aspect ratio of the bounding box that contains the iris mask is computed over time. Large values indicate blink events while small values are determined as open-eye states.

Phuong et al. [2] presented a blink detection method based only on eye landmarks. Eye Aspect Ratio (EAR) is calculated from the eye landmarks, and, based on the max and current EAR values, a dynamic threshold is applied for each participant, which determines the eye blink state.

In Ref. [3], Kuwahara et al. estimated the eye fatigue sensitivity from detected spontaneous blinks. They applied a simple moving average filter over the EAR values, then applied Eye Aspect Ratio Mapping to further reduce the noise caused by irregular movements, such as looking down. First, they classified blinks, and then a strong correlation is presented in the experimental results between the median spontaneous blink rate and the time between the objective estimation of eye fatigue and the subject’s awareness of eye fatigue.

Kraft et al. [4] calculated relative EAR, which is the current EAR divided by the maximum of the last n=2*FPS values. Then, a set of conditions must be met to select blink candidates, which are classified by applying a threshold.

### 1.2. Appearance-Based Methods

Appearance features are extracted using two branches of Deep Neural Networks (DNNs) in [5]. Convolutional layers extracted features from the eye patch; meanwhile, another DNN model extracted the characteristics of the eye patch vector and reduced the features by use of the fully connected layers directly. Given their different structures, the proposed DCNN and DNN are combined to build a deep integrated model, called DINN. A transfer learning strategy was applied to extract effective abstract eye features and improve the classification capability on small-sample datasets.

In the work of [6], cropped eyes from RGB texture were used as an input to a VGG16 trained from scratch for binary eye blink state classification. Their main contribution was the introduction of a new blink dataset called mEBAL. The samples are recorded with an Electroencephalography (EEG) band, Near Infrared (NIR), and RGB cameras, which further improve on previous datasets. The authors showed that using RGB texture without temporal patterns can lead to great performance if there are quality data to train on.

The RT-GENE dataset [7] is used for gaze estimation in natural environments, but Cortacero et al. [8] extended the pipeline, so gaze estimation and blink detection are integrated into a unified network. They also published the RT-BENE dataset, which contains cropped close eye patches from RT-GENE with eye state annotations. Their dataset covers a wider range of camera–subject distances and head poses, which is beneficial for training DNNs and led to further improving the state-of-the-art results.

EyeNet [9] is proposed for binary eye state classification. They evaluated the model under different conditions (lighting, reflection, appearances, and devices) and also show some improvements over other methods (e.g., [5]). While they have shown cross-database evaluations, we will extend this with additional databases and show how to produce better results on several databases at the same time.

In the work of [10], a robust, real-time pipeline with auxiliary models was proposed considering rotation compensation and head pose orientations. The CNN produced consistently better results than the support vector machine method in their experiments.

Recently, an additional CNN called 4D was presented in [11]. It is based on the VGG19 architecture with modified hyperparameters. The network is evaluated on the MRL dataset and presented better results than others [12,13] in the literature.

### 1.3. Motion-Based Methods

Motion vectors are applied to blink detection in [14], and then the research is extended in [15]. In the latter, motion vectors are obtained by applying Gunnar–Farneback tracking in the eye region. Eyeblink states are determined by providing a normalized average motion vector with standard deviation and time constraints to a state machine. Motion information is calculated between two frames; in later works, multiple timestamps’ representation is used instead.

In the work of [16], appearance and motion information are simultaneously utilized. Local Binary Pattern (LBP) visual descriptor is extracted to represent the local eye region. The difference between the LBPs from two consecutive frames is used to encode the motion characteristics of eye blinks. The combination of appearance and movement features had a good impact on the final performance; they are concatenated as the input of LSTM. To deal with the multiple temporal cases, a multi-scale LSTM (MS-LSTM) model was proposed, which uses the outputs of the last T LSTM units jointly by concatenation.

More recently, [17] introduced an approach that is divided into two main phases. First, face detection and eye localization are implemented. Then, within the second phase, eye blink detection is conducted via moving-windowed SVD using pixel energy, and eye blink verification is performed via a 2D Pyramidal Bottleneck Block Network (PBBN), which produces the final predictions. They presented improved performance compared to [6,16] by using temporal patterns from a simple energy-based feature.

Ref. [18] utilized a Long-term Recurrent Convolutional Network (LRCN) for eye blinking detection to expose deep fake videos. They estimated facial landmarks, and, after alignment, the eye region is cropped. The visual feature extractor is a VGG16, while the dynamic information was captured by an LSTM. While the mean resting blinking rate is 17 blinks/min or 0.283 blinks per second (https://www.ncbi.nlm.nih.gov/pubmed/9399231, accessed on 25 July 2023), during a conversation, this rate increases to 26 blinks/min and decreases to 4.5 blinks/second while reading. Their method is focused on the detection of eye blinking in the videos, which is a physiological signal that is not well presented in the synthesized fake videos. The output of the LRCN model is the predicted probability of the binary eye state.

Building upon LRCN, in [19], Eye-LRCN was proposed as a novel approach to blink detection and blink completeness detection. The Siamese architectures have proven effective for other problems with high class imbalance [20]. Since most of the eye blink databases available today are class-imbalanced, there are several improvements over the baseline model proposed by Li et al.; e.g., they applied a Siamese architecture for CNN training and they used a bidirectional LSTM instead of a unidirectional LSTM.

### 1.4. Contribution

Appearance-based methods can extract richer information than feature-based methods. The feature-based method cannot be applied if the quality of input data is not good enough, and face-, landmark-, or iris detectors fail. The method we present tries to complement the literature by combining the above categories.

In this work, we propose a fast transformer-based framework for eye blink detection that can effectively combine low- and high-level feature sequences considering several challenges, such as lighting changes, and a variety of head poses, and also utilizes motion information from sequences of the aforementioned features. We present a modified multimodal transformer with linear attention (LinMulT) [21], which considers multiple inputs, such as RGB texture, iris and eye landmarks, ear, and head pose angles. To our knowledge, this is the first work to use transformer architecture and implement an efficient fusion of input features, including head pose angles. The source code for our implementation is available (code available at https://github.com/fodorad/BlinkLinMulT, accessed on 25 July 2023).

Our contributions are listed as follows:

- We propose a novel transformer-based blink detection model called BlinkLinMulT that efficiently combines low- and high-level features from video sequences using linear complexity attention mechanisms.
- Cross-dataset evaluations are performed to quantify the robustness of BlinkLinMulT on unseen samples and it is found that a single network trained on a union of datasets improves the results obtained on all datasets separately.
- We present a feature fusion ablation study and show that the proposed method works well even on extreme head poses.
- The proposed approach is evaluated on blink presence detection and eye state recognition tasks and multiple public benchmark datasets. The results obtained are similar to or better than those provided by state-of-the-art models on the respective tasks and databases.

The rest of the paper is organized as follows: Section 2 describes the eye blink detection pipeline, the proposed backbone, and sequence models developed for our experiments. The image and video datasets used in our experiments are introduced in Section 3, and then Section 4 presents the performances of our models under different conditions and experimental settings. Finally, closing remarks and future work are outlined in Section 5.

## 2. Materials and Methods

### 2.1. Eye Feature Extraction

Utilizing the head pose information is a crucial aspect in various scenarios, particularly during conversations. The participants tend to lower their gaze toward the table or look down at themselves, causing the top eyelids to become visible. This subtle cue can be challenging for a CNN to differentiate as it operates on frames. Thus, utilizing sequence models becomes essential as they can benefit from temporal information. These cases can be detected or learned if the network has access to the head pose angles predicted by, e.g., 3DDFA_V2 [22]. Additionally, visualizing head pose angles could potentially provide valuable insights to better understand the dynamics of these sessions.

Aside from head pose estimation, 3DDFA_V2 is used for facial landmark prediction on images, and for determining the location of the eyes. The eye patches are extracted and resized to 64 × 64 pixels. For fine eye landmark prediction, we used MediaPipe Face Mesh [23], which is a residual neural network that can predict dense coordinates even if the face is partially occluded. Then, we calculated Eye Aspect Ratio (EAR) introduced in [24]. MediaPipe Iris [25] is a tiny neural network that predicts 5 landmarks in 2D: the pupil center, and 4 points of the outer iris circle. For more high-level features, we calculated the iris diameters and the distances between the pupil and the eyelid landmarks. The list of features with the corresponding dimensions is in Table 1.

### 2.2. Backbone Models

EyeNet [9] is a CNN designed for classifying eye states. It comprises convolutional, max pooling, and global average pooling layers with fully connected layers. The model performed well on datasets like CEW, ZJU, and MRL; therefore, we considered using it in our experiments.

We also incorporated ResNet50 and DenseNet121 backbones in our experiments as they were found to be the best-performing models in [8]. The aforementioned CNNs are pretrained on ImageNet.

Motivated by recent literature, we included Contrastive Language-Image Pre-Training (CLIP) models in our experiments to assess their effectiveness [26]. The utilized visual backbones are a modified ResNet50 and a Vision Transformer (ViT-B/16).

We fine-tuned and tested all backbones to determine the optimal feature extractor backbone for our proposed sequence model.

### 2.3. Proposed Method

**Figure 1:**
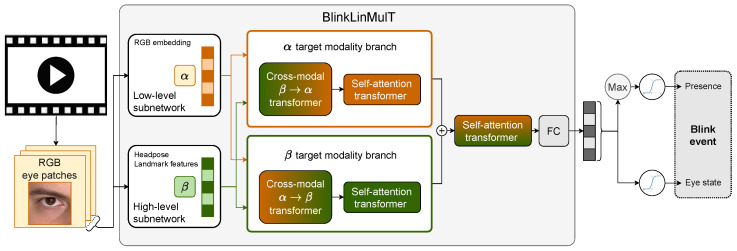
BlinkLinMulT: a multimodal transformer architecture for blink presence detection and frame-wise eye state recognition.

We propose a fast multimodal transformer, called BlinkLinMulT, shown in Figure 1, for blink presence detection and eye state recognition. The architecture is motivated by Tsai et al. [27]; however, we replaced the quadratic attention modules with linear versions [28], providing similar performance [21] while being easier to train.

Input embeddings indicated by darker striped columns can be features derived from the raw data or the outputs of a pre-trained deep model. We extracted α embeddings from the RGB texture using one of the backbone models introduced in Section 2.2. Head pose and landmarks are predicted with deep networks like 3DDFA_V2, MediaPipe Iris, and Face Mesh, while the β embeddings are generated by a subnetwork of fully connected layers.

Cross-modal transformers translate one information source (e.g., β, here associated with the landmark features) to another one (e.g., α, here associated with the RGB texture) by learning keys and values of modality β and the queries from the α modality. Both α→β and β→α are fused by an individual cross-modal transformer. A transformer network with self-attention enhances the information within the sequence branch-wise. Then, the output sequences of the modality branches are concatenated in the feature dimension and fused by a self-attention transformer. A linear attention mechanism is used within the networks to increase efficiency by reducing computational resources, i.e., time and memory.

Finally, we used a fully connected layer on top of the transformers, which is used for two tasks: (i) by only considering the maximum of the logits within the sequence, a sigmoid is applied to determine whether blinking is present in the sequence; (ii) sigmoid is applied to the frame-wise estimates to determine eye states. The outputs of the network are logits; sigmoid is applied within the loss function while training, and during inference.

### 2.4. Blink Estimation Pipeline

**Figure 2:**
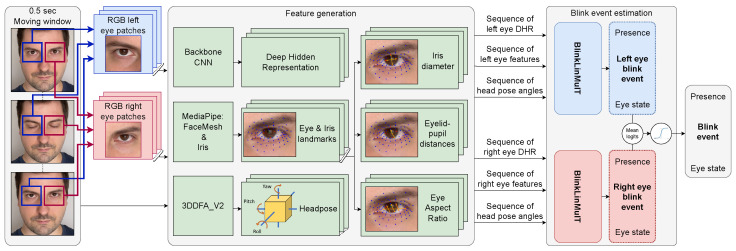
Overview of the blink estimation pipeline.

A series of steps are involved in blink estimation. Initially, the frames are extracted from the video. In our experiments, the monitored person’s face bounding box is annotated; otherwise, face detection and tracking are required. The length of a blink is 0.1–0.4 s/blink (http://bionumbers.hms.harvard.edu/bionumber.aspx?id=100706&ver=0, accessed on 25 July 2023); therefore, we chose the analyzing window to cover only 0.5 s. The 3DDFA_V2 model is applied to close face crops to obtain a sequence of head poses. Eye patches are then separately processed to extract high-level features like iris, pupil, and eye landmarks, which are used for EAR, iris diameter, and eyelid–pupil distance calculations. Furthermore, a backbone CNN extracts deep hidden representations of the RGB eye patches. These sets of features are utilized as inputs to a linear multimodal transformer that estimates blinks for each eye independently. A single blink annotation is defined for every single image; therefore, we consider estimations both from the left and right eyes. The network logits are averaged, and a sigmoid function is applied to make a final decision to obtain the final blink presence score for the sequence, and frame-wise eye state scores for determining the position of the blink event within the sequence. The components are organized into a pipeline, which can be seen in Figure 2.

## 3. Datasets

The architectures detailed in Section 2.2 have been trained and evaluated on four image datasets and five video datasets, as detailed next in Section 3.1 and Section 3.2.

### 3.1. Image Datasets

The Closed Eyes in the Wild (CEW) dataset was created by Song et al. [29]. Close face crops are collected from 2423 subjects; 1192 subjects with both eyes closed are collected directly from the Internet, and 1231 subjects with eyes open are selected from the Labeled Face in the Wild (LFW [30]) database. The face images are resized to 100 × 100 pixels. We used 3DDFA_V2 to crop both eye regions based on the landmarks, and then the patches are resized to 64 × 64 pixels. The CEW dataset does not have pre-defined training and test images; therefore, similar to others in the literature, we employed a 10-fold cross-validation (CV) scheme to measure the performance of backbone models.

Song et al. [29] cropped the original ZJU eye blink dataset [31] images to extract eye patches and expanded the initial set of images by rotation, blurring, the addition of Gaussian noise, and contrast adjustment. The original image resolution is relatively low (320 × 240 pixels), and the extracted eye patches are 24 × 24 pixels. We resized the images to 64 × 64 pixels, which is a common size among the used datasets.

The MRL Eye dataset [32] was collected from thirty-seven participants (thirty-three men and four women). This dataset contains 84,898 infrared images in low and high resolution captured by different sensors like RealSense, IDS, and Aptina. The images are captured in various lighting conditions (the annotations are “bad” and “good”) and under three reflection modes based on the size of reflections (annotations are “none”, “small”, and “high”). There are also annotations for the eye patches that are with or without glasses. The eye state property contains binary information about two eye states.

The videos on the RT-GENE dataset [7] were recorded using a Kinect v2 RGB-D camera to provide RGB images at 1920 × 1080 resolution. Eye patches are extracted from the face images and then annotated and published as RT-BENE [8]. However, to estimate landmarks around the eyes and head pose, we used the full-face images from RT-GENE and the blink annotation from RT-BENE. We denote the subset, where both the image and the annotation were available, as ${\mathrm{RT}-\mathrm{BENE}}^{img}$ in our experiments. There are 16 participants in this dataset, and, following the authors’ split, subjects with IDS 0, 14, 15, and 16 are used as the test set.

### 3.2. Video Datasets

The multimodal sequences of ${\mathrm{RT}-\mathrm{BENE}}^{seq}$ are created from ${\mathrm{RT}-\mathrm{BENE}}^{img}$. The face images with blink states, among other frame-wise features, are stacked along the time dimension to form sequences.

The EyeBlink8 dataset contains eight videos that were recorded in a home environment. Four participants are sitting in front of the camera. There are 408 eye blinks on 70,992 annotated frames with a resolution of 640 × 480.

TalkingFace is a single 200-s-long video stream, which is created to evaluate facial landmarks detection precision. The video is captured with 25 FPS and a resolution of 720 × 576, and it contains a single participant talking in front of the camera. Annotations for 61 eye blinks are also available, which were used in several related works besides their small size.

The Researcher’s Night dataset was collected during an event called Researcher’s Night 2014. People were asked to read an article on a computer screen or blink while being recorded. The authors of the dataset collected 107 videos with 223,000 frames of different people with various head poses and cluttered backgrounds. There are two subsets: Researcher’s Night 15 (RN15) and Researcher’s Night 30 (RN30) that are captured with 15 and 30 FPS with a resolution of 640 × 480.

## 4. Results and Discussion

### 4.1. Training Strategy and Evaluation Metrics

The models were fine-tuned using Adam with decoupled weight decay regularization (AdamW [33]). The initial learning rate was set to $1\times 10^{-3}$, and L2 regularization to $5\times 10^{-4}$. The learning rate is multiplied by 0.1 if no improvement is observed in the validation loss for a period of 10 epochs. Binary cross-entropy is used as the loss function between the frame-wise prediction and the corresponding ground truth for all experiments. We used 64 as the batch size. The networks are trained using a single NVIDIA RTX A4000 with 16 GB VRAM. The CNN backbones were trained for 310 epochs on the image datasets, whilst BlinkLinMulT was trained for 50 epochs using samples extracted from the video datasets. To handle the unbalanced class distribution of the datasets (Table 2), we calculated the class weights per dataset in advance, and oversampling is achieved by weighting the loss itself. Due to the unbalanced nature of the datasets, we chose the F1 score as the main performance metric; however, precision (P), recall (R), and average precision (AP) are also calculated.

### 4.2. Backbone Within-Dataset Evaluations

First, we trained and evaluated all frame-wise backbone models on the image datasets. We report the performance in Table A1.

The CEW database is relatively small in terms of the number of samples, but it is a popular benchmark dataset as most faces are frontal, balanced by class labels, and there are also higher-resolution images. All backbone models performed well on this dataset with only a slight 0.006 difference in F1 score between the worst and best models in a 10-fold cross-validation. The EyeNet results are successfully reproduced (0.990±0.006 F1 score); however, the ResNet50 and DenseNet121 outperformed it under the same conditions (0.994±0.003 and 0.995±0.002 F1 scores, respectively). The positive effect of the better pre-training method is measured with higher-resolution images using the CLIP ResNet50 and ViT-B/16; the models achieved the highest average scores with 0.996±0.005 and 0.996±0.003.

The backbones performed worse on low-resolution, augmented, and noisy images of the ZJU database. The two best-performing models are the ResNet50 and DenseNet121 with 0.929 and 0.927 F1 scores, as well as 0.965 and 0.959 AP, respectively. CLIP backbone variants like ViT-B/16 performed worse (with 0.915 F1 score and 0.957 AP) due to there being less spatial context available to learn global dependencies, and a large number of parameters can lead to overfitting on small datasets. CNNs can effectively learn local features with fewer parameters, making them a better choice for smaller-resolution images.

The ${\mathrm{RT}-\mathrm{BENE}}^{img}$ dataset contains more samples, rich in different head poses, but it is rather unbalanced, with many more open-eye samples than blinking samples, and the method that fills the glasses frame might introduce additional noise. The difficulty of the database is reflected in the accuracy of the models; EyeNet achieved 0.868, followed by the CLIP ResNet50 and ViT-B/16 with 0.870 and 0.871 F1 scores, respectively. DenseNet121 performed well with 0.883, but ResNet50 outperformed the other models by a considerable margin with a 0.912 F1 score and 0.946 AP.

### 4.3. Eye Patch Resolution Dependence

CLIP backbones are underperforming, and one possible reason for this is the resizing of low-resolution images from 64 × 64 to 224 × 224. While the information content is the same, the larger input makes pattern recognition more difficult. To confirm this assumption, we have trained a ResNet50 with the same hyperparameters, the only difference being in the image dimensions, and it is denoted as ResNet50-224px in Table A1. The CNN trained on the 224 × 224 images achieved lower performance metric scores in every case versus its 64 × 64 counterpart: 0.929 vs. 0.932 precision, 0.837 vs. 0.893 recall, 0.881 vs. 0.912 F1 score, and 0.918 vs. 0.946 AP.

### 4.4. Backbone Cross-Dataset Evaluations

We also evaluated the performance of the backbone models on unseen samples. First, all backbone models are trained on one of the datasets, then evaluated on the rest without further fine-tuning. Metrics are calculated considering all combinations. The metrics of cross-dataset evaluations are in Table A2. When the networks trained on the ZJU dataset and evaluated on others, the F1 score is acceptable on the test set of the ZJU dataset, e.g., 0.929 for the best-performing model ResNet50; the same network can only achieve 0.652 on the CEW and 0.502 on the ${\mathrm{RT}-\mathrm{BENE}}^{img}$, showing a massive performance degradation, 30%, and 46%, respectively. The networks in general learned better features when trained on the CEW images (e.g., F1 score of ResNet50 is 0.717 on ZJU, 0.672 on ${\mathrm{RT}-\mathrm{BENE}}^{img}$); however, the best cross-dataset performance can be achieved when the ${\mathrm{RT}-\mathrm{BENE}}^{img}$ is used as a training dataset. ResNet50 and DenseNet121 outperformed the other backbones by a large margin; however, we cannot choose a clear best model based on this experiment; 0.806 and 0.851 F1 scores are achieved on ZJU, while 0.894 and 0.836 F1 scores on CEW, respectively.

As the samples of the different datasets are produced in a similar way, they differ mainly only in diversity. Therefore, we also trained the models on the union of the datasets, and report the metrics in Table A3. DenseNet121 utilized the extended set of samples and achieved the best F1 scores among other metrics over all backbones. Training on the union of the datasets improved on previous results measured in Section 4.2: the F1 scores changed from 0.995 to 0.997 on CEW, from 0.927 to 0.933 on ZJU, and from 0.883 to 0.913 ${\mathrm{RT}-\mathrm{BENE}}^{img}$.

To verify the robustness of the DenseNet121 model, we measure the performance using the MRL images under different conditions: one condition is whether or not the samples have glasses, whether or not there are good or bad light conditions, and the level of distracting reflection on the eye surface or glasses. F1 scores from four experiments are summarized in Table 3. For both Exp-A and Exp-B, DenseNet121 has trained on the union of the CEW, ZJU, and ${\mathrm{RT}-\mathrm{BENE}}^{img}$ datasets, but, for Exp-A, we evaluated on the whole MRL dataset, and, for Exp-B, we used the test subset of MRL, the images belonging to participants 35 and 36. The difference between the average F1 scores of the experiments is negligible; therefore, for Exp-C and Exp-D, we used the same holdout set. For Exp-C, we also added samples from the MRL dataset to the previous CEW, ZJU, and ${\mathrm{RT}-\mathrm{BENE}}^{img}$ triplet. Finally, Exp-D in the last column was evaluated for ease of comparison with methods in the literature and for completeness; in this case, DenseNet121 was only trained and tested on the MRL database with a 0.75–0.15–0.15 train–validation–test split; a 0.9953 average F1 score is successfully reproduced. We observed a 3.8% degradation if we are not fine-tuning the network using the MRL images, and also the F1 score is increased by 2% if the MRL train set is added to the union of the training databases. We used the DenseNet121 with the weights obtained in Exp-C as the RGB backbone model within the proposed BlinkLinMulT.

### 4.5. BlinkLinMulT Within-Dataset Evaluations

The proposed BlinkLinMulT predicts frame-wise eye state scores, which are also used for determining the position of the blink event within the sequence, and then the blink presence is determined and results for both tasks are reported in Table 4. The accuracy of the blink presence estimate is better, and there may be two reasons for this: one is that, in many cases, the start of the blink event is annotated as “closed eye” even though the person’s eye is still open in the given frames. While the model correctly estimates “open eye” for these frames, it may appear as an error in the table. On the other hand, although the annotation is defined frame-wise, the decoders available today do not extract the frames in exactly the same way, especially if there are different FPS within the video. In these cases, some frame slips may occur, which we have not corrected manually.

### 4.6. BlinkLinMulT Cross-Dataset Evaluations

Similar to Section 4.4, we have carried out cross-dataset evaluations using the proposed BlinkLinMulT model. We used the EyeBlink8, ${\mathrm{RT}-\mathrm{BENE}}^{seq}$, RN15, and RN30 datasets for training and evaluation, and, due to its limited size, the TalkingFace is used only for testing purposes. Table 5 is grouped by test databases for ease of reference; we experienced improved performance when RN15 and RN30 datasets are used within training despite their lower resolution and in-the-wild difficulty.

In Ref. [8], their hypothesis that increased training sample size leads to an increase in performance is accepted, while the performance barely increased by using 100% of the RT-BENE training data instead of 75%. We extend it with a hypothesis that increased diversity in training samples—in terms of resolution, head pose angles, and the participants’ biomarkers—leads to an increase in performance; we trained the BlinkLinMulT on the union of the datasets. Table 6 shows that the eye state recognition and blink presence detection performance are consistently improved compared to those experiments, where only a single dataset is used for training. BlinkLinMulT captured the unique patterns from all used datasets; therefore, the eye state recognition F1 score increased from 0.723 to 0.87 for EyeBlink8, 0.792 to 0.871 for RN15, and 0.792 to 0.836 for RN30. The blink presence detection F1 score increased from 0.938 to 0.959 for RN15 and 0.921 to 0.933 for RN30. While, in the literature, the proposed models are retrained for each dataset, our network is trained once and performs well on all the public blink benchmark datasets.

### 4.7. Comparison to the Literature

We show that our method generalizes well to different scenarios, and the performance of our network is comparable to or surpasses the performance of state-of-the-art methods, in Table 7. Note that TalkingFace is only used for testing, but, nevertheless, the looking-down events are successfully recognized, while other methods make mistakes. The proposed BlinkLinMulT excels in the blink presence detection task on all datasets. The per-frame evaluation metrics of the eye state recognition task are slightly lower; however, this is due to occasional frame-shifting during decoding and the fact that annotation often starts and ends with an open eye. In these cases, although the network is not flawed, it will be reflected in the frame-wise results during evaluation.

#### 4.7.1. Feature Significance

To measure the added value of the different features used in our experiments, we trained deep networks using the input features separately and then using the different combinations. The F1 scores obtained on the image test subsets are in Table 8, and we also report the metric using the video datasets in Table 9. To facilitate transparency, the following feature groups are created:

- MediaPipe Iris landmark features (ILM): the feature set contains the iris landmarks, iris diameters, and eyelid–pupil distances.
- MediaPipe Face Mesh landmark features (FLM): the feature set contains the eye landmarks and eye aspect ratio.
- Head pose angles (HP): the head pose angles are highlighted independently.
- Texture (T): RGB texture contains the most information.

Based on the dimensionality of the feature set, different models are used. We evaluated multiple hyperparameters for the networks, and report the best possible F1 metrics, that could be obtained on the test subset of ${\mathrm{RT}-\mathrm{BENE}}^{img}$ datasets in Table 8. For ILM and FLM, a fully connected shallow network is trained. For T, the same DenseNet121 is used, reported in Table A3. T+ILM+FLM utilizes every available high-level feature. Head pose angles are excluded because ZJU and MRL datasets are used for training the CNN backbone; however, it contains only eye patches, and head pose angles are not available.

The RGB texture is the richest in detail; therefore, increased performance is expected in this case. While the iris and eye landmark features alone show fairly low performance, combining them with the texture further improves the performance on ${\mathrm{RT}-\mathrm{BENE}}^{img}$ compared to texture-only evaluation (0.922 vs. 0.913 F1 scores).

We evaluated the effectiveness of individual and combined modalities on all video datasets. F1 score is reported in Table 9 for the eye blink presence task. The HP, ILM, and FLM sequences are fed to a linear self-attention transformer, denoted as LinT. ILM and FLM landmark-based feature sets show potential when the dynamics are also utilized by a sequence model. For sequences of RGB frames, the uni-modal version of our proposed model, BlinkLinT, is used: LinT is learning the dynamics of DenseNet121 hidden representations. The model achieved high F1 scores on multiple datasets, showing the importance of low-level RGB texture over high-level features.

While the head pose angles do not hold information about the blinking event, the combination with the texture and other high-level features helps in extreme cases. Our proposed model, BlinkLinMulT, utilizes both low- and high-level information sources effectively. There are negligible differences between the different versions of the proposed model, but we would like to highlight that, in the last case, where all feature sequences are used, the problematic extreme blinks when the person looks down are detected in TalkingFace, achieving a 1.0 F1 score in the blink presence detection task.

#### 4.7.2. Frame-Wise Model versus Sequence Model

To show the importance of modeling the dynamics of motion, we evaluated our best backbone model, DenseNet121 (Table A3), per frame on sequences of the video datasets. Then, similar to the BlinkLinMulT pipeline, we performed max aggregation within the sequence to determine the presence of blinking for both eyes. In Table 9, the transformer-based sequence modeling shows superiority over frame-wise estimations.

#### 4.7.3. Head Pose Angle Dependence

**Figure 3:**
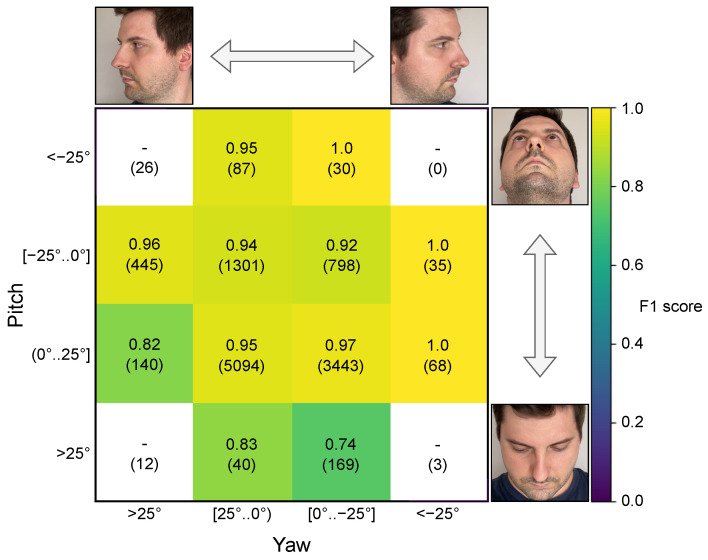
Head pose angle dependence of BlinkLinMulT in the case of blink presence detection task. The head poses are predicted by 3DDFA_V2; the colors represent the F1 score calculated for the blink presence task, which is also written within the boxes together with the number of samples (in parenthesis) considered during the metric evaluations. F1 score cannot be calculated for those extreme cases, where closed-eye samples are not available. Test samples from all 5 sequence datasets are used for the experiment. Blinks can be predicted accurately in the case of frontal faces, and while the participant is looking up. Performance slightly decreases when the monitored person is looking down.

We investigated the impact of head pose on the blink presence detection performance of BlinkLinMulT and report the F1 scores in Figure 3 and Accuracy, AP F1 scores in Table A4. The union of ${\mathrm{RT}-\mathrm{BENE}}^{seq}$, EyeBlink8, RN15, RN30 test subsets, and the samples from the TalkingFace video is used to include more diverse head poses. For each sequence, the means of frame-wise head pose estimations are calculated. The result of the experiment revealed that the performance of the model drops at higher yaw angles compared to the frontal faces; the 0.917 F1 score is calculated from 633 blink samples for >$25^{\circ }$ angles compared to the 0.951 average F1 score from 10,962 frontal samples. In these cases, self-occlusion makes the prediction harder for the network, which originated from the fact that, beyond the imbalanced class distribution, only a fraction of the blink patterns have a high head pose angle associated with them. In the case of positive pitch angles, the participants are looking down. Most of the errors originated from this extreme head pose because the eyelids are more visible, they move over a shorter distance, and the landmark-based features are less precise considering the available resolution. We experienced a lower F1 score compared to negative pitch angles (0.744 vs. 0.933). Higher performance metrics are expected for samples when the monitored people are looking up; it is quite common for the camera to be positioned slightly below the facial lines. Even with the low number of extreme samples (9%), the performance is fairly consistent and acceptable across head pose variations.

## 5. Conclusions

We propose a transformer-based model for efficient eye blink detection, called BlinkLinMulT. Specifically, we modified the multimodal transformer with linear attention (LinMulT), which fuses multiple inputs, such as RGB texture, iris and eye landmarks features, and head pose angles.

Our contributions include the following: we propose BlinkLinMulT, a novel transformer-based blink detection model that efficiently combines low- and high-level features from video sequences using linear complexity attention mechanisms. We performed cross-dataset evaluations to assess the robustness of BlinkLinMulT on unseen samples, demonstrating that a single network trained on a union of datasets improves results across all datasets individually. We showed how increased eye patch dimensionality of low-resolution eye patches degrades the performance of current SOTA backbones, e.g., CLIP ViT-B/16 dependence, and also presented the difference in performance when the motion dynamics are utilized compared to frame-wise evaluations that ignore such an important relationship between frames. We present a feature significance ablation study and showcase the effectiveness of our proposed method under extreme head poses. Our results demonstrate comparable or superior performance compared to state-of-the-art models on blink presence detection and eye state recognition tasks, using public benchmark databases.

By leveraging the power of transformers for an efficient fusion of input features, our model achieves accurate blink detection during challenging in-the-wild scenarios. The promising results obtained on public benchmark datasets highlight the potential of our approach for advancing the field of blink detection and its applications in various facial analysis tasks.

## Figures and Tables

**Table 1:** Features used in the experiments. The second and third column indicate the inputs from which the given feature was calculated and the dimensions of the features.

Feature Name	Source	Dimension
RGB eye patch	RGB face crop	(64, 64, 3)
Head pose angles	RGB face crop	(3,)
Face Mesh landmarks	RGB eye patch	(72, 2)
Iris landmarks	RGB eye patch	(5, 2)
Iris diameters	Iris landmarks	(2,)
Eyelid-pupil distances	Iris and Face Mesh landmarks	(2,)
Eye Aspect Ratio	Face Mesh landmarks	(1,)

**Table 2:** Overview of the datasets.

Type	Dataset	# Samples	Samples for Training and Validation		Samples for Testing		% Blink in Test Set
			# Open Eyes	# Blinks	# Open Eyes	# Blinks	
Images	CEW	2423	1050	1009	182	182	50
	ZJU	8984	5770	1574	1230	410	25
	MRL	84,898	34,618	33,830	8334	8116	49
	${\mathrm{RT}-\mathrm{BENE}}^{img}$	107,169	72,281	2847	30,347	1694	4
Sequences	${\mathrm{RT}-\mathrm{BENE}}^{seq}$	6969	4356	547	1869	197	10
	EyeBlink8	4616	3422	293	786	115	13
	RN15	6439	2830	434	2903	272	9
	RN30	11,231	5346	650	4729	506	10
	TalkingFace	324	-	-	263	61	19

**Table 3:** Performance of the DenseNet121 backbone under different settings. Exp-A: DenseNet121 is trained on the union of the image datasets (ZJU, CEW, ${\mathrm{RT}-\mathrm{BENE}}^{img}$) and evaluated on the full MRL dataset. Exp-B: the union of the image datasets is used for training, and only the MRL test set is used for evaluating the network. Exp-C: the union of image datasets is expanded with the MRL train and validation images, and the MRL test set is used for measuring the performance. Exp-D: the network is trained using the MRL train and validation samples, and results are calculated on the MRL test set.

Condition	F1			
	Exp-A	Exp-B	Exp-C	Exp-D
With glasses	0.9518	0.9632	0.9797	0.9959
Without glasses	0.9714	0.9478	0.9742	0.9944
Good light	0.9679	0.9723	0.9838	0.9953
Bad light	0.9631	0.9469	0.9749	0.9948
No reflection	0.9727	0.9560	0.9784	0.9954
Low reflection	0.9613	0.9536	0.9934	1.0000
High reflection	0.8987	0.9574	0.9486	0.9912
Average	0.9553	0.9567	0.9761	0.9953

**Table 4:** Eye state recognition and blink presence detection performances of the proposed method BlinkLinMulT on the ${\mathrm{RT}-\mathrm{BENE}}^{seq}$, EyeBlink8, RN15, and RN30 datasets.

Task	Dataset	*p*	R	F1
Eye state	${\mathrm{RT}-\mathrm{BENE}}^{seq}$	0.940	0.931	0.936
	EyeBlink8	0.569	0.990	0.723
	RN15	0.692	0.927	0.792
	RN30	0.683	0.886	0.771
Presence	${\mathrm{RT}-\mathrm{BENE}}^{seq}$	0.922	0.959	0.940
	EyeBlink8	0.991	1.000	0.996
	RN15	0.925	0.952	0.938
	RN30	0.916	0.927	0.921

**Table 5:** Eye state recognition and blink presence detection performance of the proposed method BlinkLinMulT during cross-dataset evaluation using the EyeBlink8, ${\mathrm{RT}-\mathrm{BENE}}^{seq}$, RN15, RN30, and TalkingFace datasets.

Train DB	Test DB	$P_{\mathrm{state}}$	$R_{\mathrm{state}}$	${F1}_{\mathrm{state}}$	$P_{\mathrm{presence}}$	$R_{\mathrm{presence}}$	${F1}_{\mathrm{presence}}$
EyeBlink8	${\mathrm{RT}-\mathrm{BENE}}^{seq}$	0.139	0.462	0.213	0.313	0.746	0.441
RN15	${\mathrm{RT}-\mathrm{BENE}}^{seq}$	0.337	0.592	0.430	0.676	0.731	0.702
RN30	${\mathrm{RT}-\mathrm{BENE}}^{seq}$	0.295	0.480	0.365	0.811	0.721	0.763
${\mathrm{RT}-\mathrm{BENE}}^{seq}$	EyeBlink8	0.958	0.679	0.795	0.950	0.983	0.966
RN15	EyeBlink8	0.531	0.954	0.682	0.983	0.974	0.978
RN30	EyeBlink8	0.704	0.967	0.815	0.983	0.974	0.978
${\mathrm{RT}-\mathrm{BENE}}^{seq}$	RN15	0.797	0.464	0.586	0.858	0.868	0.863
EyeBlink8	RN15	0.838	0.787	0.811	0.919	0.838	0.877
RN30	RN15	0.834	0.807	0.820	0.941	0.930	0.935
${\mathrm{RT}-\mathrm{BENE}}^{seq}$	RN30	0.851	0.571	0.683	0.908	0.860	0.883
EyeBlink8	RN30	0.655	0.768	0.707	0.873	0.804	0.837
RN15	RN30	0.404	0.890	0.555	0.819	0.905	0.860
${\mathrm{RT}-\mathrm{BENE}}^{seq}$	TalkingFace	0.834	0.490	0.617	0.968	1.000	0.984
EyeBlink8	TalkingFace	0.961	0.805	0.876	1.000	0.984	0.992
RN15	TalkingFace	0.947	0.614	0.745	1.000	0.967	0.983
RN30	TalkingFace	0.994	0.714	0.831	1.000	1.000	1.000

**Table 6:** Eye state recognition and blink presence detection performance of the proposed method BlinkLinMulT on the test subsets of TalkingFace, ${\mathrm{RT}-\mathrm{BENE}}^{seq}$, EyeBlink8, RN15, and RN30 datasets. The model is trained on the union of the train subsets of these datasets.

Task	Train DB	Test DB	P	R	F1
Eye state	Union	${\mathrm{RT}-\mathrm{BENE}}^{seq}$	0.824	0.796	0.810
	Union	EyeBlink8	0.782	0.979	0.870
	Union	RN15	0.867	0.876	0.871
	Union	RN30	0.890	0.787	0.836
	Union	TalkingFace	0.993	0.614	0.759
Presence	Union	${\mathrm{RT}-\mathrm{BENE}}^{seq}$	0.914	0.965	0.938
	Union	EyeBlink8	0.991	0.991	0.991
	Union	RN15	0.977	0.941	0.959
	Union	RN30	0.966	0.901	0.933
	Union	TalkingFace	1.000	1.000	1.000

**Table 7:** Comparison of our BlinkLinMulT to the methods in the literature in the case of two tasks: blink presence detection and frame-wise eye state recognition. The highest F1 scores per dataset and task are shown in bold.

Database	Task	Method	*p*	R	F1
TalkingFace	Presence	[2]	0.951	0.936	0.943
		[10]	-	-	0.950
		[4]	0.983	0.934	0.958
		[34]	-	-	0.971
		[19]	-	-	0.979
		Ours	1.000	1.000	**1.000**
EyeBlink8	Eye state	[35]	-	-	0.834
		[19]	-	-	0.910
		[8]	0.995	0.958	**0.976**
		Ours	0.782	0.979	0.870
	Presence	[10]	-	-	0.870
		[4]	0.909	0.904	0.905
		[34]	-	-	0.913
		[19]	-	-	0.946
		[24]	0.943	0.960	0.952
		[2]	0.953	0.958	0.955
		Ours	0.991	0.991	**0.991**
RN	Eye state	[19]	-	-	0.807
		[8]	-	-	**0.913**
		Ours	0.878	0.828	0.852
	Presence	[34]	-	-	0.879
		[19]	-	-	0.906
		Ours	0.970	0.915	**0.942**
RT-BENE	Eye state	[35]	-	-	0.529
		[19]	-	-	0.602
		[8]	0.664	0.791	0.721
		Ours	0.824	0.796	**0.810**

**Table 8:** Blink presence detection performance ablation study, presenting the impact of different features. The networks trained on the union of CEW, MRL, and ${\mathrm{RT}-\mathrm{BENE}}^{img}$ datasets. The metric is the F1 score on the individual test subsets. The highest F1 score is shown in bold.

Modality	Model	${\mathrm{RT}-\mathrm{BENE}}^{\mathrm{img}}$
ILM	Dense	0.453
FLM	Dense	0.595
T	DenseNet121	0.913
T+ILM+FLM	DenseNet121+Dense	**0.922**

**Table 9:** Blink presence detection performance ablation study, presenting the impact of different features. The networks trained on the union of ${\mathrm{RT}-\mathrm{BENE}}^{seq}$, EyeBlink8, RN15, and RN30 datasets. The metric is the F1 score on the individual test subsets. The highest F1 scores per dataset are shown in bold.

Modality	Model	${\mathrm{RT}-\mathrm{BENE}}^{\mathrm{seq}}$	EyeBlink8	RN15	RN30	TalkingFace
HP	LinT	0.267	0.227	0.303	0.276	0.529
ILM	LinT	0.814	0.978	0.869	0.860	0.992
FLM	LinT	0.761	0.974	0.925	0.867	1.000
T	DenseNet121	0.779	0.887	0.645	0.620	0.976
T	BlinkLinT	0.922	0.991	0.946	0.920	0.992
T+HP	BlinkLinMulT	**0.962**	**0.991**	**0.973**	0.929	0.992
T+FLM+ILM	BlinkLinMulT	0.947	**0.991**	0.958	**0.939**	0.992
T+HP+FLM+ILM	BlinkLinMulT	0.938	**0.991**	0.959	0.933	**1.000**

## Data Availability

Publicly available datasets were analyzed in this study. These data can be found here: TalkingFace, EyeBlink8, Researcher’s Night-https://www.blinkingmatters.com/research (accessed on 25 July 2023), RT-BENE-https://github.com/Tobias-Fischer/rt_gene (accessed on 25 July 2023).

## Appendix A

### Appendix A.1. Backbone Within-Dataset Evaluations

**Table A1:** Performance of the backbone models on the CEW, ZJU, and ${\mathrm{RT}-\mathrm{BENE}}^{img}$ datasets. 10-fold cross-validation is performed on the CEW dataset, while, in the case of the ZJU and ${\mathrm{RT}-\mathrm{BENE}}^{img}$, we evaluated on the test set. The highest F1 score is in bold, and the second highest score is underlined.

Database	Backbone	*p*	R	F1	AP
CEW	EyeNet	0.992 ± 0.007	0.987 ± 0.009	0.990 ± 0.006	0.999 ± 0.001
	ResNet50	0.994 ± 0.008	0.993 ± 0.005	0.994 ± 0.003	0.999 ± 0.001
	DenseNet121	0.998 ± 0.003	0.993 ± 0.005	0.995 ± 0.002	0.999 ± 0.001
	CLIP ResNet50	0.996 ± 0.006	0.996 ± 0.004	0.996 ± 0.005	0.999 ± 0.001
	**CLIP ViT-B/16**	0.996 ± 0.006	0.997 ± 0.004	**0.996 ± 0.003**	0.999 ± 0.001
ZJU	EyeNet	0.908	0.939	0.923	0.943
	**ResNet50**	0.929	0.929	**0.929**	0.965
	DenseNet121	0.938	0.917	0.927	0.959
	CLIP ResNet50	0.901	0.883	0.892	0.941
	CLIP ViT-B/16	0.950	0.883	0.915	0.957
${\mathrm{RT}-\mathrm{BENE}}^{img}$	EyeNet	0.878	0.858	0.868	0.930
	**ResNet50**	0.932	0.893	**0.912**	0.946
	ResNet50-224px	0.929	0.837	0.881	0.918
	DenseNet121	0.891	0.876	0.883	0.926
	CLIP ResNet50	0.908	0.835	0.870	0.932
	CLIP ViT-B/16	0.927	0.821	0.871	0.933

### Appendix A.2. Backbone Cross-Dataset Evaluations

**Table A2:** Performance of the backbone models using cross-dataset evaluation. The highest F1 ratings and corresponding models are in bold. The second highest ratings and corresponding models are underlined.

Model	Train DB	Test DB	*p*	R	F1	AP
EyeNet	CEW	ZJU	0.749	0.766	0.758	0.847
ResNet50	CEW	ZJU	0.687	0.749	0.717	0.813
DenseNet121	CEW	ZJU	0.636	0.759	0.692	0.791
CLIP-ResNet50	CEW	ZJU	0.806	0.742	0.773	0.868
CLIP-ViT-B/16	CEW	ZJU	0.819	0.705	0.758	0.857
EyeNet	${\mathrm{RT}-\mathrm{BENE}}^{img}$	ZJU	0.683	0.766	0.722	0.822
ResNet50	${\mathrm{RT}-\mathrm{BENE}}^{img}$	ZJU	0.817	0.795	0.806	0.852
**DenseNet121**	${\mathrm{RT}-\mathrm{BENE}}^{img}$	ZJU	0.880	0.824	**0.851**	0.936
CLIP-ResNet50	${\mathrm{RT}-\mathrm{BENE}}^{img}$	ZJU	0.807	0.654	0.722	0.820
CLIP-ViT-B/16	${\mathrm{RT}-\mathrm{BENE}}^{img}$	ZJU	0.676	0.693	0.684	0.775
EyeNet	ZJU	CEW	0.433	1.000	0.604	0.499
ResNet50	ZJU	CEW	0.540	0.822	0.652	0.668
DenseNet121	ZJU	CEW	0.694	0.866	0.771	0.825
CLIP-ResNet50	ZJU	CEW	0.474	0.968	0.636	0.565
CLIP-ViT-B/16	ZJU	CEW	0.452	0.930	0.608	0.549
EyeNet	${\mathrm{RT}-\mathrm{BENE}}^{img}$	CEW	0.811	0.784	0.797	0.859
**ResNet50**	${\mathrm{RT}-\mathrm{BENE}}^{img}$	CEW	0.881	0.907	**0.894**	0.950
DenseNet121	${\mathrm{RT}-\mathrm{BENE}}^{img}$	CEW	0.792	0.885	0.836	0.903
CLIP-ResNet50	${\mathrm{RT}-\mathrm{BENE}}^{img}$	CEW	0.693	0.880	0.775	0.793
CLIP-ViT-B/16	${\mathrm{RT}-\mathrm{BENE}}^{img}$	CEW	0.662	0.830	0.733	0.740
EyeNet	ZJU	${\mathrm{RT}-\mathrm{BENE}}^{img}$	0.177	0.217	0.195	0.116
ResNet50	ZJU	${\mathrm{RT}-\mathrm{BENE}}^{img}$	0.459	0.554	0.502	0.497
DenseNet121	ZJU	${\mathrm{RT}-\mathrm{BENE}}^{img}$	0.636	0.680	0.657	0.666
CLIP-ResNet50	ZJU	${\mathrm{RT}-\mathrm{BENE}}^{img}$	0.098	0.452	0.161	0.098
CLIP-ViT-B/16	ZJU	${\mathrm{RT}-\mathrm{BENE}}^{img}$	0.132	0.619	0.217	0.124
EyeNet	CEW	${\mathrm{RT}-\mathrm{BENE}}^{img}$	0.529	0.845	0.651	0.497
ResNet50	CEW	${\mathrm{RT}-\mathrm{BENE}}^{img}$	0.555	0.852	0.672	0.514
DenseNet121	CEW	${\mathrm{RT}-\mathrm{BENE}}^{img}$	0.624	0.801	0.701	0.740
**CLIP-ResNet50**	CEW	${\mathrm{RT}-\mathrm{BENE}}^{img}$	0.737	0.834	**0.782**	0.781
CLIP-ViT-B/16	CEW	${\mathrm{RT}-\mathrm{BENE}}^{img}$	0.559	0.782	0.652	0.558

### Appendix A.3. Backbone Union Evaluations

**Table A3:** Performance of the backbone models using the union of multiple datasets. The highest F1 ratings and corresponding models are in bold. The second highest ratings and corresponding models are underlined.

Model	Train DB	Test DB	P	R	F1	AP
EyeNet	Union	CEW	0.974	0.955	0.965	0.995
ResNet50	Union	CEW	0.994	0.987	0.990	0.999
**DenseNet121**	Union	CEW	0.994	1.000	**0.997**	0.999
CLIP-ResNet50	Union	CEW	0.899	0.905	0.902	0.961
CLIP-ViT-B/16	Union	CEW	0.969	0.981	0.975	0.993
EyeNet	Union	ZJU	0.912	0.861	0.886	0.948
ResNet50	Union	ZJU	0.920	0.900	0.910	0.961
**DenseNet121**	Union	ZJU	0.914	0.954	**0.933**	0.956
CLIP-ResNet50	Union	ZJU	0.819	0.859	0.838	0.899
CLIP-ViT-B/16	Union	ZJU	0.872	0.917	0.894	0.957
EyeNet	Union	${\mathrm{RT}-\mathrm{BENE}}^{img}$	0.913	0.901	0.907	0.954
ResNet50	Union	${\mathrm{RT}-\mathrm{BENE}}^{img}$	0.879	0.879	0.879	0.947
**DenseNet121**	Union	${\mathrm{RT}-\mathrm{BENE}}^{img}$	0.913	0.913	**0.913**	0.959
CLIP-ResNet50	Union	${\mathrm{RT}-\mathrm{BENE}}^{img}$	0.851	0.800	0.825	0.899
CLIP-ViT-B/16	Union	${\mathrm{RT}-\mathrm{BENE}}^{img}$	0.939	0.850	0.892	0.944

### Appendix A.4. Head Pose Angle Dependence of the Proposed BlinkLinMulT

**Table A4:** Blink presence prediction performance of the proposed BlinkLinMulT. Test samples from all 5 sequence datasets are used for the experiment. Yaw and pitch degrees are predicted by 3DDFA_V2. Extreme yaw and pitch angles, when the participant is looking sideways or down, might cause slightly decreased performance.

Orientation	Angle	Accuracy	AP	F1
Yaw	<$-25^{\circ }$	0.991	0.990	0.960
	$\left[-25^{\circ }..0^{\circ }\right)$	0.990	0.985	0.956
	$\left[0^{\circ }..25^{\circ }\right)$	0.990	0.985	0.945
	>$25^{\circ }$	0.984	0.954	0.917
Pitch	<$-25^{\circ }$	0.986	0.996	0.933
	$\left[-25^{\circ }..0^{\circ }\right)$	0.985	0.973	0.930
	$\left[0^{\circ }..25^{\circ }\right)$	0.992	0.988	0.957
	>$25^{\circ }$	0.951	0.800	0.744
